# Foreign body in the Eustachian tube - case presentation and technique used for removal

**DOI:** 10.1016/S1808-8694(15)30764-3

**Published:** 2015-10-19

**Authors:** Fernando de Andrade Quintanilha Ribeiro

**Affiliations:** 1 Adjunct Professor - Department of Otorhinolaryngology - School of Medical Sciences - Santa Casa de São Paulo

**Keywords:** foreign body, middle ear, eustachian tube

## Abstract

Foreign bodies of the external ear are very common; the same can not be said about foreign bodies of the middle ear, especially of the Eustachian tube.

**Case presentation:**

Alcoholic and psychopathic patient presented a foreign body (barbecue wooden stick) purposefully introduced in his middle ear and Eustachian tube during an act of delinquency. The foreign body was stuck in the tube and could not be removed externally. It was surgically removed as described by the authors later on in the paper.

**Comments:**

The patient recovered well, with no sequela on the facial nerve and without important vertigo.

## INTRODUCTION

Foreign bodies in the ear are frequent, and they are almost always located in the external auditory meatus^1^^,^^2^. The most common in children are seeds and parts of plastic toys. Button batteries have also been reported and may cause more problems if not removed early on^3^. Middle ear foreign bodies are rarer and usually happen after accidents, and because of their consequences, they are diagnosed quickly^4^. We may consider as foreign bodies those objects used in surgeries such as ossicle prostheses, plastic tapes used to avoid tympanic adherenses or ventilation tubes^5^. This hereby described case of a foreign body in the Eustachian tube is extremely rare.

## LITERATURE REVIEW

We did not find any report of a foreign body in the Eustachian Tube reported in the literature.

## CASE PRESENTATION

**Figure 1:**
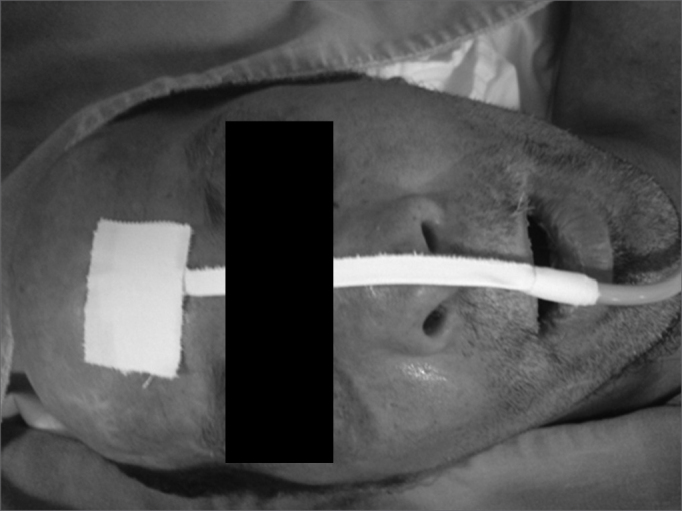
Multiple injuries patient.

**Figure 2:**
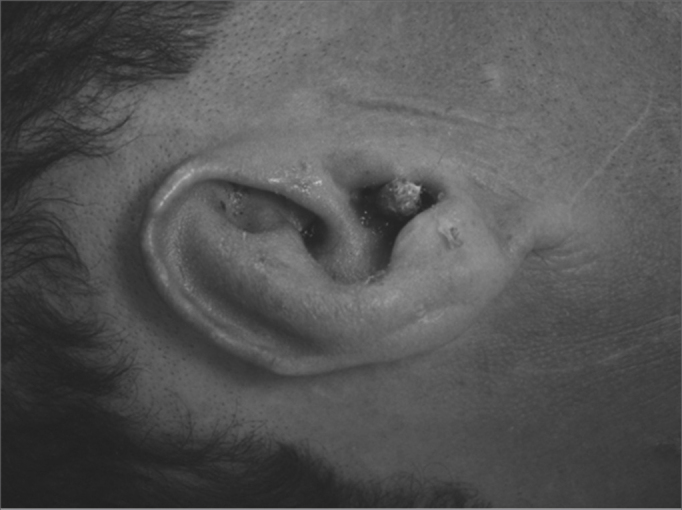
Foreign body in the right side external acoustic meatus.

**Figure 3:**
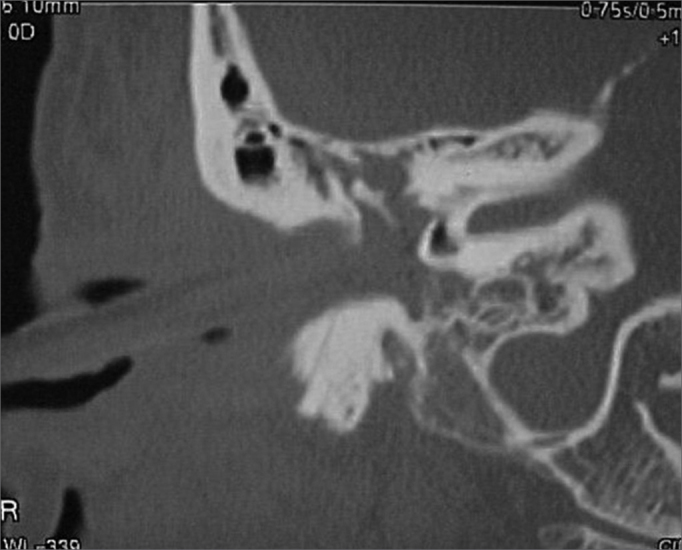
Foreign body in the external acoustic meatus and middle ear, dislocating the ossicular chain towards the mastoid region.

**Figure 4:**
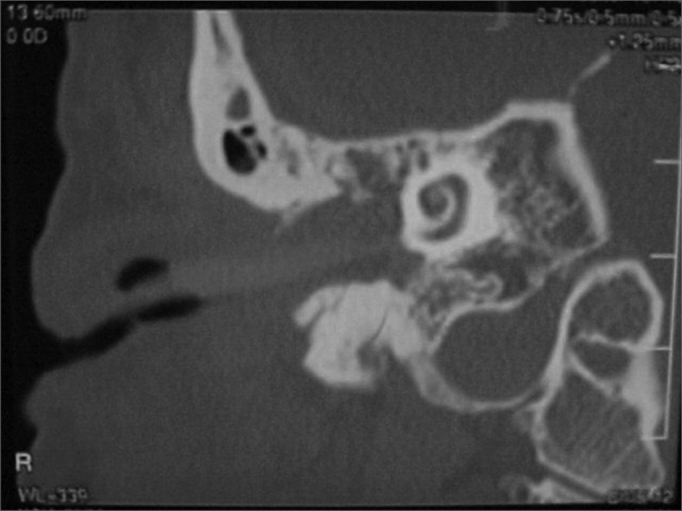
Foreign body filling up the middle ear.

**Figure 5:**
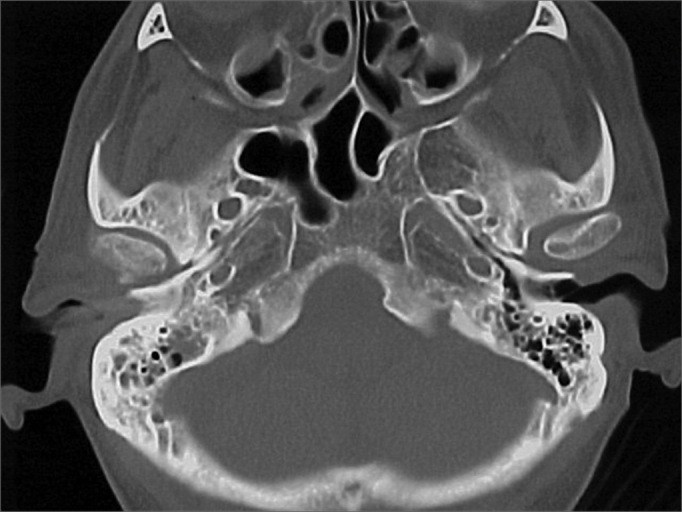
Foreign body in the right Eustachian Tube, compared to the left tube.

A 50 year old male, street dweller, chronic alcoholic, demented, was admitted to the hospital with multiple cranio-facial injuries, among them he had an ethmoidal fracture, small laminar subdural hematoma, internal carotid thrombosis on the right side and a foreign body that could be seen in the right external acoustic meatus (Figure 1, Figure 2). After being discharged from observation in the neurology ward he was refferred to the ENT department, where the CT scan showed a foreign body that had penetrated through the external acoustic meatus, invaded the middle ear and went down the Eustachian tube. There was an injury in the carotid canal and thrombosis in the internal carotid artery (Figure 3, Figure 4, Figure 5, Figure 6, Figure 6a, Figure 7, Figure 7a), in its furcation, proven by arteriogram (Figure 8, Figure 9, Figure 9a). The patient refused a complete audiologic workup, and we noticed only a profound hearing loss on the right side. Internal medicine doctors examined him and aproved him for general anesthesia for foreign body removal. He was conscious and able to converse, however because of his dementia he did not make sense when he spoke.

**Figure 6:**
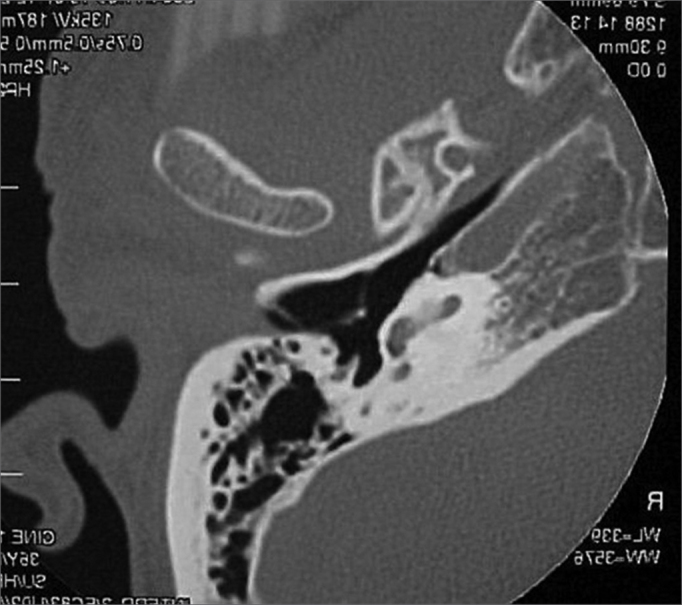
View of the left tube, with the CT scan for comparison.

**Figure 6a:**
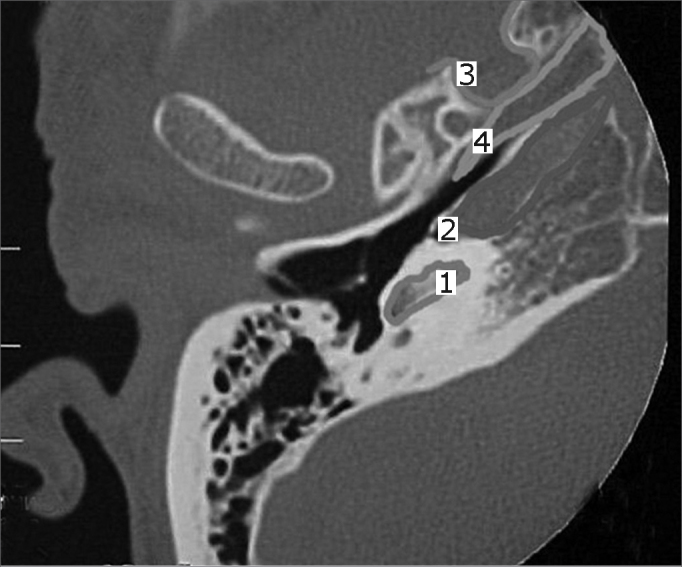
Blue - cochlea in red - carotid canal in green - central nervous system in yellow - part of the tube filled by the foreign body.

**Figure 7:**
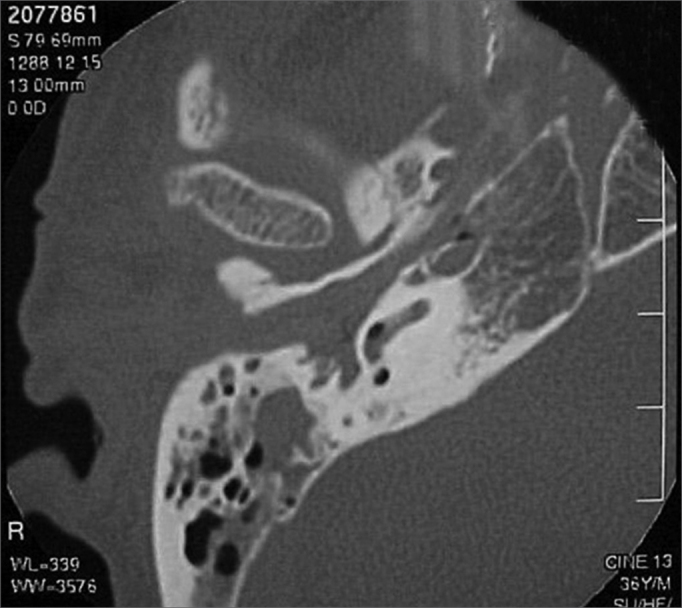
View of the right side Eustachian tube, filled by the foreign body.

**Figure 7a:**
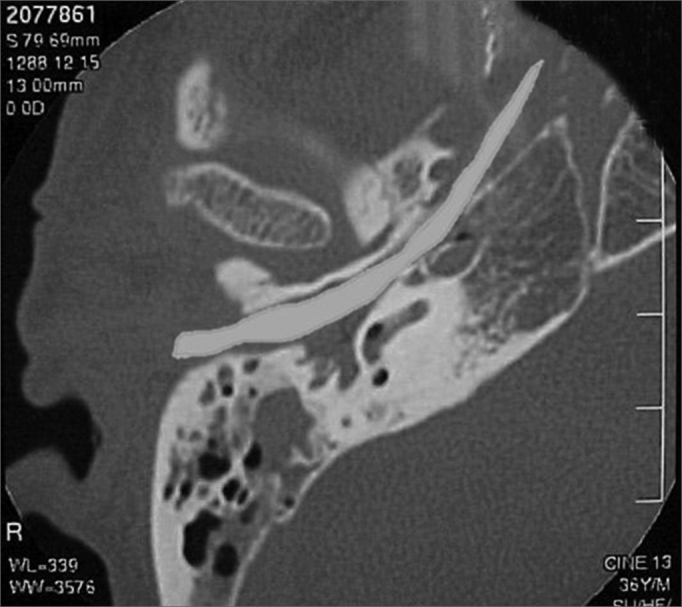
Foreign body schematics to enhance view.

**Figure 8:**
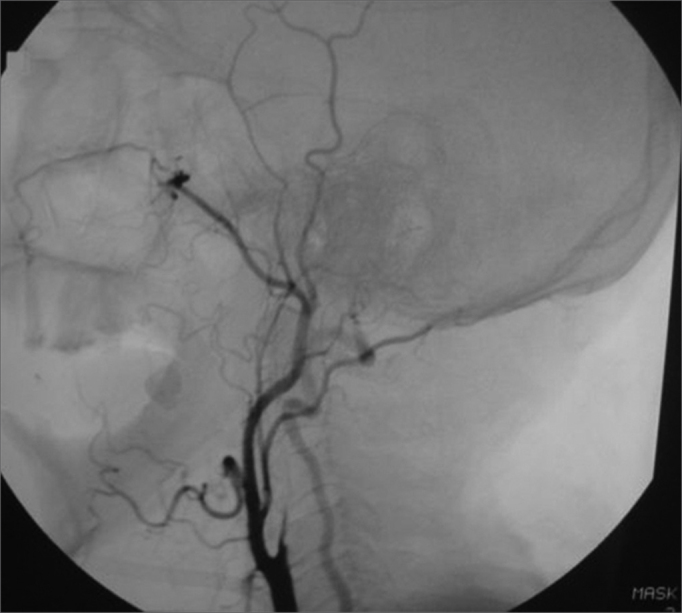
Side view arteriogram, noticing the thrombosis blocking the entire right side carotid artery.

**Figure 9:**
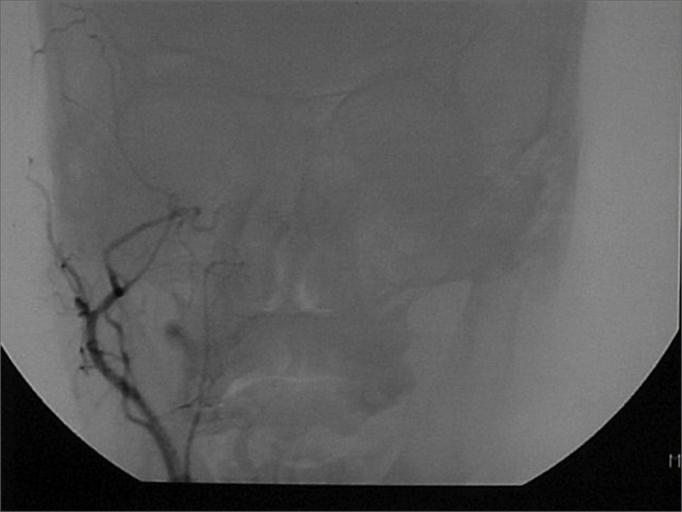
Coronal view with thrombosis blocking the entire right carotid artery.

**Figure 9a:**
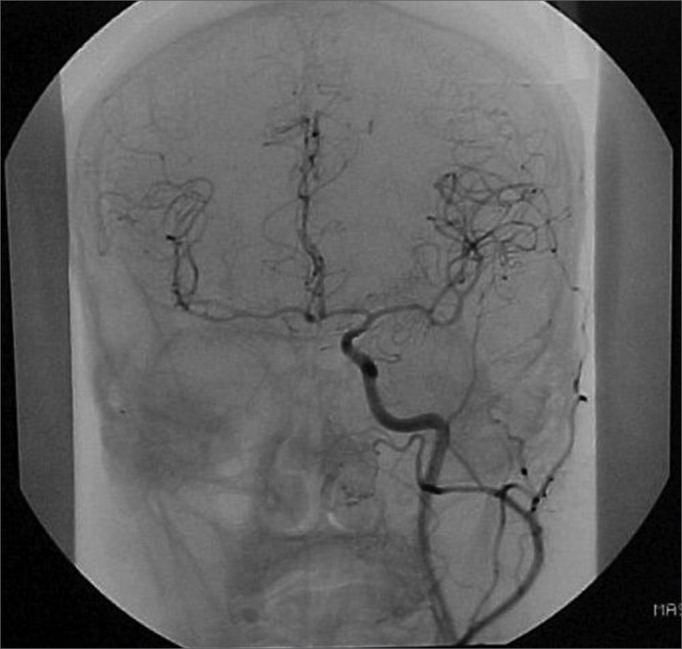
Left carotid coronal view to compare and the circulation at the Willis polygon.

## SURGERY DESCRIPTION

**Figure 10:**
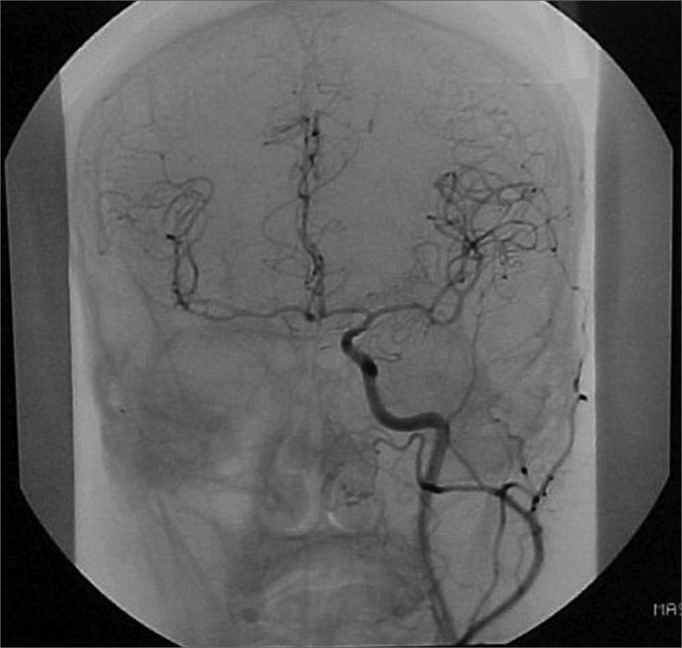
Postoperative cavity, similar to a canal wall down procedure.

**Figure 11:**
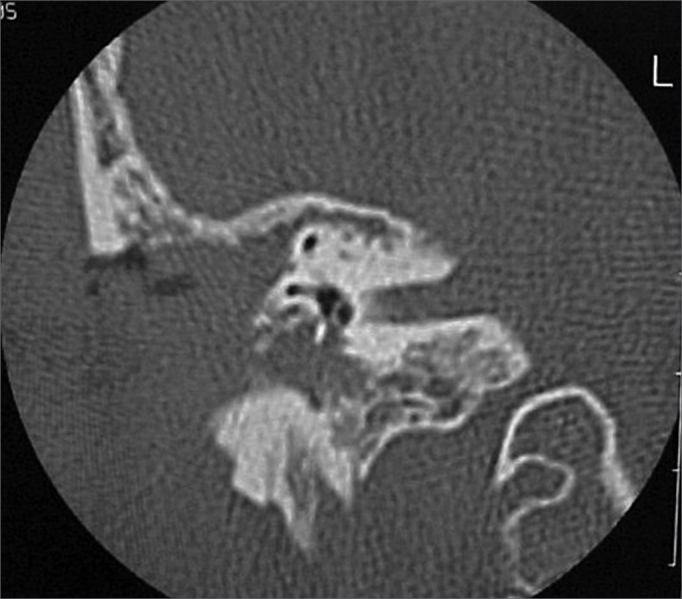
Postoperative cavity, notice lesion in the inferior semicircular canal.

**Figure 12:**
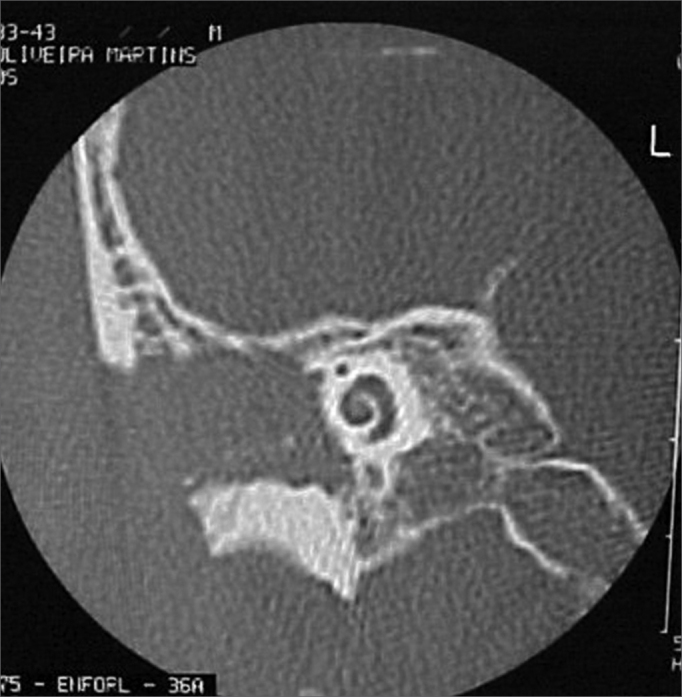
Postoperative cavity with intact cochlea.

**Figure 13:**
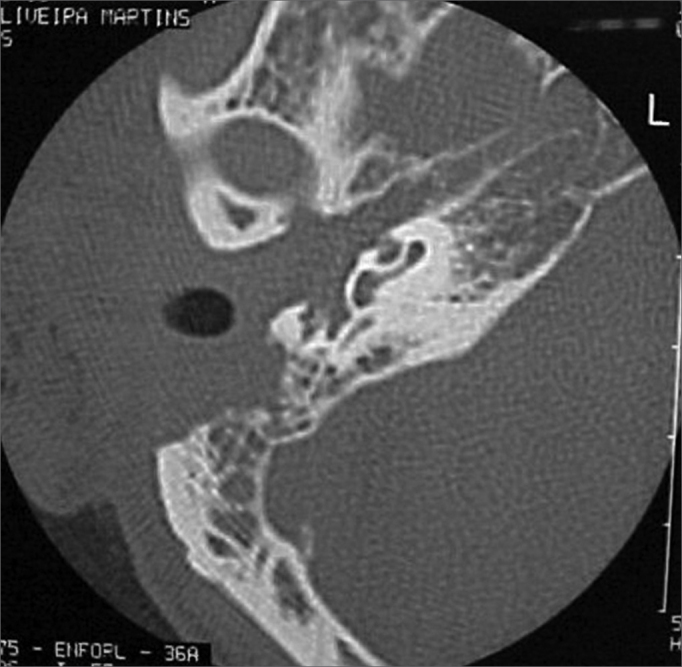
Eustachian tube region before the procedure, still with the foreign body.

**Figure 14:**
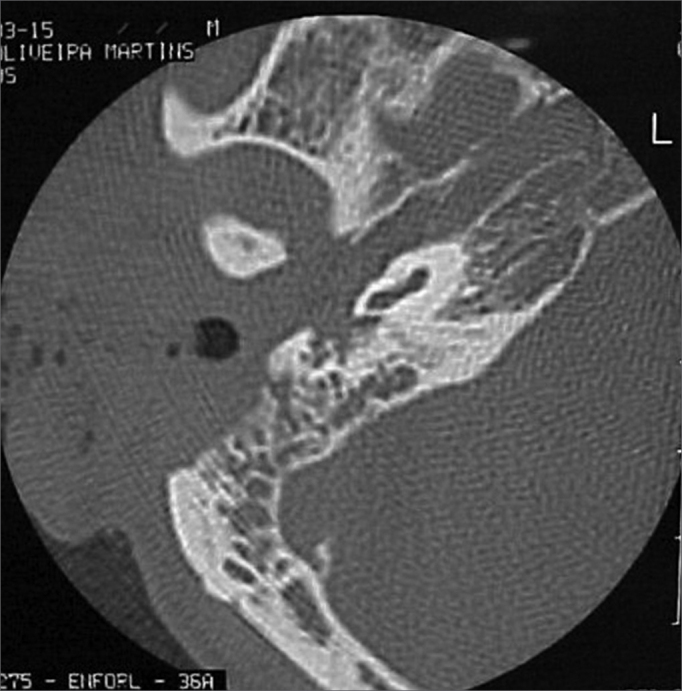
Enlarged tube after the procedure for foreign body removal.

Under general anesthesia we noticed a foreign body that appeared to be a wood stick used to make barbecue. With a strong forceps we mobilized it in an attempt to remove it, and because of the carotid thrombosis it would be very unlikely to have profuse bleeding. The foreign body did not move. We did a retro-auricular approach with incision in the meatus skin. Since it filled the whole space, we did a mastoidectomy, through which we noticed incus dislocation towards the mastoid cavity. It was removed, together with the malleus. We did a canal wall down approach and in the mastoid cavity we noticed that the wood stick filled the entire middle ear and seemed to progress towards the Eustachian tube. We tried to remove it again, unsuccesfully. We decided to cut it with the burr and keep on using the burr all the way to the begining of the Eustachian Tube. We did not see the stapes. At this point we noticed that the stick penetrated the tube like a locking wedge used in ship building. With the burr we proceeded in cutting all the bone around but especially the wood stick itself which was cut from inside and then pushed inside the lumen made by the burr. There were no reference points, but we saw the carotid canal erosion. We progressed like this for about six centimeters, always drilling down on the stick itself and removing a little of the adjacent bone. Our attempts to remove it were unsuccesful. After this course we noticed the bone had ended and there was soft tissue ahead (cartilaginous portion of the tube). At this point the tip of the wood stick felt loosen and could be removed. Medially we observed a small central nervous system dehiscence. Under the surgical microscope we observed a larger space that would open inferiorly and that was later confirmed as being the nasopharynx after we used the nasal endoscope to look at it. The broad cavity left behind in the tube was filled with muscle and the surgical wound was similar to that left after a canal wall down mastoidectomy (Figure 10, Figure 11, Figure 12, Figure 13, Figure 14). We rebuilt the meatus and sutured the retroauricular incision.

In a later CT scan we noticed na erosion of the inferior semicircular canal and of the carotid canal, and part of the central nervous system exposure.

## DISCUSSION

We did not find any similar case of foreign body in the Eustachian tube in the literature so as to compare characteristics and discuss removal approaches.

The patient's past history was uknown to us, and his interview did not add much. What we did know was that he had suffered multiple injuries from a criminal act. It was not possible to define the date of his injuries, but analyzing the internal carotid thrombosis, without brain lesion detected at the CT Scan and without aparent sequelae, it was reasonable to infer that it had not been very recent. The foreing body, a wood stick used in barbecues was probably hammered down his ear, breaking through the ear drum, hitting the promontory and finding its way throught the Eustachian Tube. It fit there under pressure like a locking wedge, without much possibility for removal. The technique we used, drilling down the foreign body, which we have used before to remove bullets, proved to be the most adequate at the time. There was not much bleeding and the patient recovered well, without facial paralysis or vertigo. He had no auditory complaints, but his audiologic evaluation was impaired by his dementia, we observed a profound hearing loss on the right side, already present in the preoperative period. He did not complain of tinnitus, but he said he could hear voices. He was later referred to the psychiatric ward and the social services.

## FINAL COMMENTS

This case shows us the extent of human violence, but also the extent of our body's endurance.
